# Rethinking Schizophrenia and Depression Comorbidity as One Psychiatric Disorder Entity: Evidence From Mouse Model

**DOI:** 10.3389/fnins.2020.00115

**Published:** 2020-03-11

**Authors:** Chunhua Zhou, Dezhi Kong, Xiaodong Zhu, Wei Wu, Rong Xue, Gongying Li, Yong Xu, Sha Liu, Hongjun Tian, Chuanjun Zhuo

**Affiliations:** ^1^Department of Pharmacology, The First Hospital of Hebei Medical University, Shijiazhuang, China; ^2^Two-Photon In Vivo Imaging Centre, Institute of Chinese Integrative Medicine, Hebei Medical University, Shijiazhuang, China; ^3^Tianjin Neurological Institute, Tianjin Medical University General Hospital, Tianjin, China; ^4^Institute of Brain Micro Scale Imaging Centre, School of Mental Health, Jining Medical University, Jining, China; ^5^MDT Center for Cognitive Impairment and Sleep Disorders, First Hospital/First Clinical Medical College of Shanxi Medical University, Taiyuan, China; ^6^Psychiatric-Neuroimaging-Genetics-Comorbidity Laboratory (PNGC_Lab), Tianjin Mental Health Centre, Mental Health Teaching Hospital of Tianjin Medical University, Tianjin Anding Hospital, Tianjin, China

**Keywords:** schizophrenia, depression, mouse model, behavioral phenotypes, prefrontal cortex neuronal activity, antipsychotic treatment

## Abstract

Schizophrenia is frequently accompanied by depressive symptoms, but the pathological mechanisms remain to be elucidated. In this study, we used chronic unpredicted mild stress plus MK801 injection to generate a mouse model of schizophrenia with depression, in which *in vivo* 2-photon calcium imaging and electrophysiological recordings were performed in conjunction with behavioral phenotyping. Compared to mice models with classical depression or to schizophrenia models, the animal models with schizophrenia and depression comorbidity presented worse psychotic and depressive symptoms. These behavioral deficits are associated with impaired neuronal calcium activities in the frontal cortex and thalamic nuclei. Moreover, in sharp contrast to classical models that have a satisfactory response to antipsychotic or antidepressant drugs, this novel schizophrenia with depression model is resilient to combined drug treatment in terms of behavioral and functional recovery. Taken together, these data indicate that schizophrenia with depression likely involves a unique pathophysiology that is different from schizophrenia or depression alone.

## Introduction

It is estimated that up to 80% of patients with schizophrenia experience depressive episodes at least once during the early phase of the disease (Upthegrove et al., 2010). Depressive disorder prevalence in patients with schizophrenia can be as high as 40% and may be observed in any disease stage (Conley et al., 2007; Fusar-Poli et al., 2013). In the past, the presence of mood syndromes was recognized as a positive prognostic indicator in patients with schizophrenia (Craddock and Owen, 2010). Recent studies, however, argue against such opinions by stating that these comorbidities predict worse schizophrenia prognoses (Siris, 2000; Yung et al., 2007; Upthegrove et al., 2010; Gardsjord et al., 2016; Helfer et al., 2016). To date, the neural mechanism underlying schizophrenia with depression remains largely unknown. Some studies argue for the impairment of the cortico-limbic circuit as the one underlying neuropsychiatric comorbidity (Totterdell, 2006), and a few studies suggest the role of neuroinflammation in schizophrenia and depression (Anticevic et al., 2015). However, the detailed mechanism, such as the activity of the neural circuit, in such a comorbid condition is unclear yet, probably due to the lack of animal models to mimic this condition. Therefore, the animal study for schizophrenia with depression should provide valuable information for clinical diagnosis and intervention.

The existence of depressive syndromes in patients with schizophrenia largely complicates the clinical remediation and, more importantly, compromises the precise definition and categorization of mental illnesses. For example, the comorbid depressive and hypomanic symptoms in schizophrenia largely affect the Diagnostic and Statistical Manual of Mental Disorders (Fifth Edition) definitions of schizophrenia and schizoaffective disorders (Siris, 2000; Helfer et al., 2016; Yung et al., 2007; American Psychiatric Association [APA], 2013). On the other hand, it becomes more difficult to adopt precise medications in schizophrenia patients who present depressive syndromes as complications. Hence, there is an urgent need to investigate the neural mechanisms underlying comorbidities between depression and schizophrenia to establish treatment targets and biomarkers, covering aspects from behavioral symptoms to neural circuit activity, to improve the medication efficiency of these patients (Bosanac and Castle, 2013; Dauwan et al., 2016; Krupchanka and Katliar, 2016; Nasrallah et al., 2015; Mao and Zhang, 2015; Upthegrove et al., 2017).

To date, clinicians have investigated depressive disorder and schizophrenia comorbidities in terms of epidemiology (Strauss and Gold, 2012; Bosanac and Castle, 2013), clinical manifestations (Strauss and Gold, 2012; Upthegrove et al., 2017; Krynicki et al., 2018), therapeutic effects (Barnes et al., 1989; Addington et al., 1996; Sandhu et al., 2013), functional outcomes (Beck, 1970; Birchwood et al., 2005; Upthegrove et al., 2014; Kam et al., 2015; McGinty et al., 2018), biological functions (Anderson et al., 2013; Khandaker et al., 2014; Samsom and Wong, 2015), somatic comorbidity (Upthegrove et al., 2018; Gundogdu et al., 2019; Postolache et al., 2019), suicide risk, and life quality (Pompili et al., 2007; Patel et al., 2014; Upthegrove, 2015; van Rooijen et al., 2019). These observations, however, do not consider the depressive symptoms in schizophrenia patients based on a unified theory and tend to isolate depressive disorder from schizophrenia. In fact, depressive disorders, as comorbid symptoms in schizophrenia, have unique features compared to those in unipolar or bipolar depressive disorders (Bosanac and Castle, 2013; Pompili et al., 2007). Therefore, the idea of recognizing depression and schizophrenia as two independent mental disorders that coexist as comorbid forms may be premature and lacks sufficient support. Such classical views should be challenged in a philosophical sense, as stated by Silverstein et al. who called for a revolutionary re-thinking of scientific questions (Hesse, 1971; O’Donohue et al., 2003).

In this study, we adopted a unified view to recognize schizophrenia with depression as one unique psychiatric disorder entity which shows distinct pathogenic mechanisms compared to models of schizophrenia or depression. More importantly, we also asked if the combined treatment with antipsychotics and antidepressants can relieve the symptoms of such schizophrenia with depression. Our hypothesis diverges from classical views stating that schizophrenia with depression syndromes are simply the combined pathologies of those two psychiatric disorders by stating unique neural mechanisms. To investigate our theory, we generated a mouse model of schizophrenia with depression and utilized two-photon *in vivo* imaging and electrophysiological recordings to study the neural activities in such model.

## Materials and Methods

### Animals and Overall Experimental Designs

**FIGURE 1:**
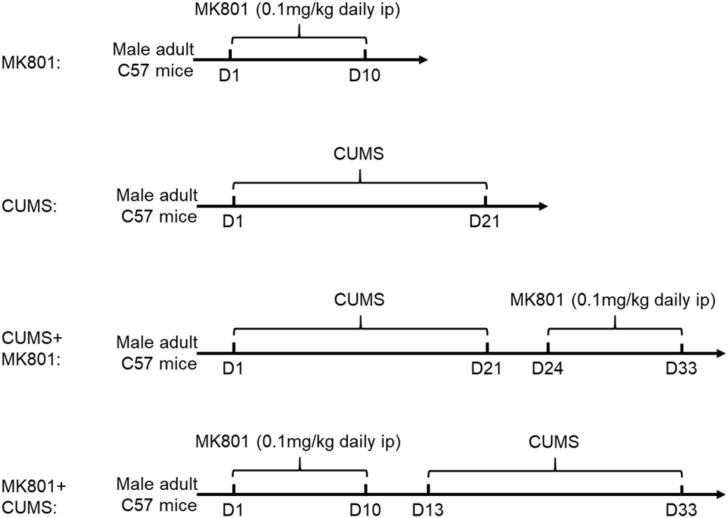
Flowchart of experimental procedures. Chronic MK801 infusion or CUMS protocol was applied to induce schizophrenia or depression model, respectively. CUMS and MK801 treatment were sequentially applied in generating depression plus schizophrenia, or schizophrenia with depression model.

Male C57BL/6 mice (5–6 weeks old) were housed in an animal facility with food and water given *ad libitum*. All animals were randomly assigned into five groups: control, MK801, chronic unpredicted mild stress (CUMS), CUMS + MK801, and MK801 + CUMS. For the control group, the mice were housed in their homecage with no intervention. To create the schizophrenia mouse model, the mice received an intraperitoneal injection of MK-801, also known as dizocilpine, a non-competitive N-methyl-D-aspartic acid (NMDA) receptor antagonist. The drug infusion lasted for 10 days, with daily dosages at 0.1 mg/kg. A CUMS model was used to create a rodent depression model as previously reported (Liu W. et al., 2018). In brief, the animals were exposed to different stressors including cage tilting, wet bedding, forced swimming, sleep deprivation, and physical restraint for 3 weeks. In the CUMS + MK801 group, representing depression with schizophrenia, the animals received MK801 at 3 days following the conclusion of the CUMS treatment. In the MK801 + CUMS model, representing schizophrenia with depression, the mice first received an MK801 injection and were enrolled in the CUMS paradigm at 3 days post-injection. The brief experimental procedure was summarized in Figure 1. The animal study was reviewed and approved by the Animal Ethical Committee of Tianjin Medical University in accordance with the Institutional Animal Care and Use Committee guidelines for animal research.

### Behavioral Phenotyping

All behavioral tasks were performed on day 3 after the end of the intervention. The animals were sequentially enrolled in a sucrose preference test and a forced swimming task with a 24-h interval between them. A second cohort of mice was tested using the prepulse inhibition (PPI) apparatus. The sucrose preference test and forced swimming tasks followed previously published methodologies (Yankelevitch-Yahav et al., 2015; Liu M. et al., 2018). For the PPI test, a 120-dB (40 ms) startle (PA) was applied after a 20-ms prepulse (PP) at 75 dB, with a time interval of 100 ms. The background noise was controlled at 65 dB. The inter-trial time was set at 30 s. Generally, three sessions were used and the scores were averaged. The PPI was calculated as (PA - PP)/(PA).

### *In vivo* Calcium Recordings

To record the prefrontal cortex (PFC) neuronal activity, previously published methods (Koukouli et al., 2017) were used with slight modifications. In brief, the anesthetized mice were fixed, and a chronic cranial window was created. Then, 200 nl of AAV2/9-syn-GCaMP6s virus (2 × 10^13^ genome copies/ml; University of Pennsylvania Vector Core) was injected bilaterally into the prelimbic cortex using the following coordinates: +2.8 mm from the bregma, ±0.5 mm. The imaging window was covered with a circular coverslip, and the skull was sealed using dental cement. A customized steel bar was embedded into the skull to enable the head of the mouse to be fixed during the imaging session.

During two-photon *in vivo* imaging, previously reported approaches (Koukouli et al., 2017) were followed. A two-photon microscope (LSM780; Zeiss, Germany) was used with a × 16, 0.8 N.A. water-immersed objective. Using an excitation wavelength of 950 nm, time-series images were recorded at 1.96 Hz for 150-s periods. The captured images were analyzed using ImageJ (National Institutes of Health, Bethesda, MD, United States). Regions of interest were selected manually in ImageJ with a FIJI plug-in package (Koukouli et al., 2017), followed by the detection and normalization of calcium transients.

### Patch-Clamp Recordings

Electrophysiological recordings were performed in thalamic medium spiny neurons following previously published protocols (Ding et al., 2010). In brief, the brain slices were prepared and infused with artificial cerebrospinal fluid (2 mM KCl, 0.12 M NaCl, 2 mM MgSO4, 1.2 mM KH2PO4, 26 mM NaHCO3, 2.5 mM CaCl2, and 11 mM glucose) at room temperature for recovery. Whole-cell patch-clamp recordings were performed to detect miniature excitatory post-synaptic currents (mEPSCs) in the presence of TTX and picrotoxin (Sigma, St. Louis, MO, United States). Pipettes for voltage-clamp recordings were filled with internal solution (all in mM: 120 CsMeSO_3_, 15 CsCl, 8 NaCl, 10 TEA-Cl, 10 HEPES, 2–5 QX-314, 0.2 EGTA, 2 Mg-ATP, and 0.3 Na-GTP; pH 7.3). Data filtered at 2–5 kHz were recorded using a MultiClamp 700A.

### Recording of Visual-Evoked Potentials

Visual-evoked potentials were measured in all groups of mice from the thalamic nuclei using previously published protocols (Montesano et al., 2015). The mice were deeply anesthetized with sevoflurane. The skull was exposed, and an electrode was placed in the visual thalamus using a stereotaxic apparatus and fixed to the skull. During the recording, the animal was placed in a dark environment, and white light stimuli (0.1 Hz, 300 ms; LED light) were presented. The signals were amplified and filtered at 1 kHz.

### Statistical Analysis

All experimental data are presented as the mean ± standard error of the mean unless otherwise specified. A two-sample Student’s *t*-test or non-parametric K-S test was used to compare the means between two groups. For multi-group comparisons, one-way analysis of variance was performed, followed by Tukey’s *post hoc* comparison. GraphPad Prism 7.0 was used for statistical analyses and data plotting.

## Results

### Distinct Behavioral Features in Mouse Models of Schizophrenia With Depression

**FIGURE 2:**
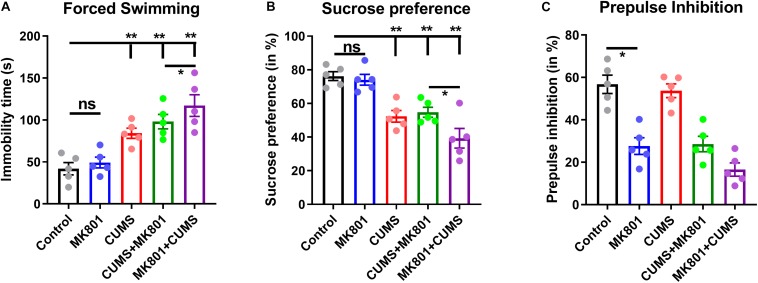
Evaluation of the depressive and the psychotic symptoms among the different rodent models. **(A)** Immobility time during the forced swimming task is shown. **(B)** The sucrose preference ratio reveals prominent anhedonia in all models except for the MK801 administration alone. **(C)** The prepulse inhibition test shows auditory gating deficits in schizophrenia but not in depression models. CUMS, chronic unpredicted mild stress. ns, no significant difference; *P < 0.05; **P < 0.01; *N* = 5 per group.

To investigate the neuropathology of schizophrenia with depression, its behavioral phenotypes were first compared with those of other commonly used depression or schizophrenia models. NMDA receptor antagonist MK801 and CUMS were combined to generate a murine model of depression with schizophrenia (CUMS + MK801) and another model that mimics schizophrenia with depression (MK801 + CUMS). To test the validity of these models, behavioral tasks examining symptoms of depression and schizophrenia were employed. The forced swimming task was used to evaluate helplessness in the rodents, although the MK801-treated mice presented minor changes, while the CUMS and CUMS + MK801 groups displayed significantly longer durations of immobility (Figure 2A). More importantly, the MK801 + CUMS group exhibited an even higher mean immobility time compared to that in the CUMS group (Figure 2A), suggesting the worsening of helplessness under these comorbid conditions. Similar patterns of anhedonia were found in the mice as well; while both the CUMS and CUMS + MK801 groups showed a decreased sucrose preference, the MK801 + CUMS mice exhibited an even lower sucrose preference rate (Figure 2B). These two datasets clearly illustrate that schizophrenia with depression normally presents more severe behavioral symptoms compared to unipolar depression. It is further noticed that although a previous report indicates an anti-depressant effect of single MK801 in a mouse CUMS model (Yang et al., 2018), our CUMS + MK801 group did not have any significant improvement of depressive phenotypes. Such discrepancy is probably due to the sub-chronic MK801 infusion used in the current study, in contrast to the single injection in the anti-depressant work. Furthermore, an evaluation of sensory gating function abnormalities, a typical symptom of schizophrenia, showed that the MK801 + CUMS group presented the most severe deficits in the auditory response among all groups, as suggested by the lower PPI ratio (Figure 2C). These data collectively demonstrate that schizophrenia with depression exhibits multiple symptoms including helplessness, anhedonia, and sensory gating deficits; most of these characteristics are further deteriorated compared to those in depression and schizophrenia alone.

### Distinct Behavioral Features in Mouse Models of Schizophrenia With Depression

**FIGURE 3:**
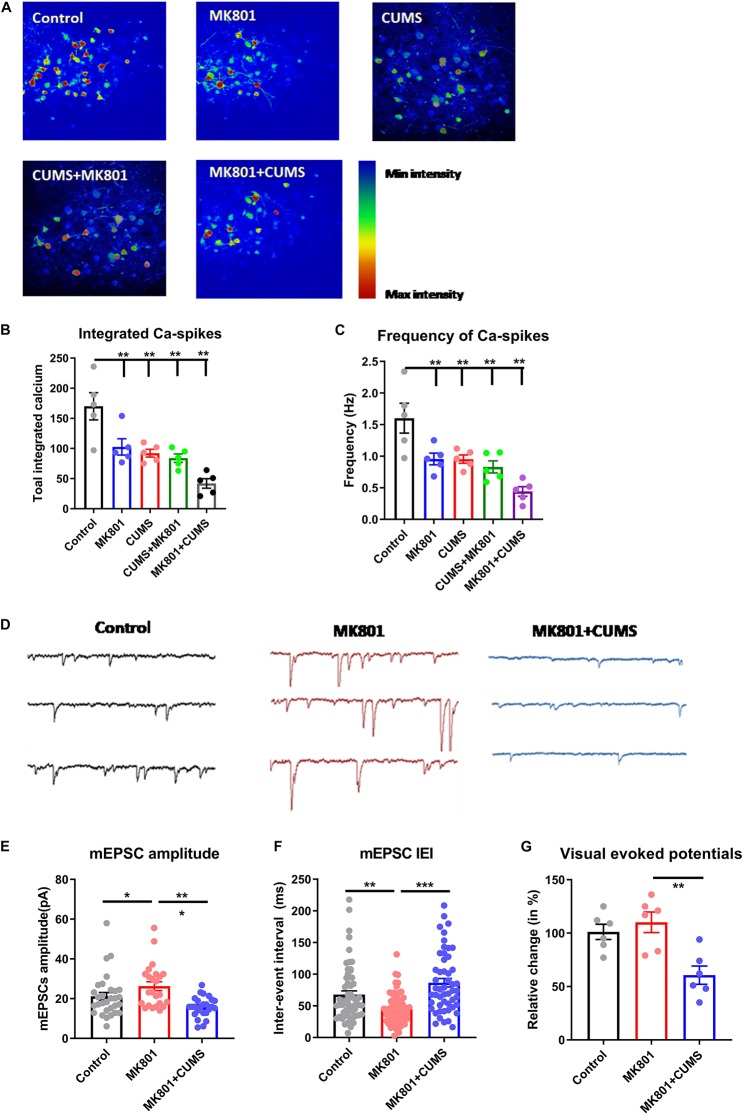
Impaired transmission in the cortical and the subcortical neural circuits. **(A)** Representative time-lapse images of calcium transients in the dlPFC in all experimental groups are shown. **(B)** Quantitative analysis results of integrated calcium spikes over the recoding period (2.5 min). **(C)** The average frequency of calcium spikes in Hz is shown. **(D)** Representative electrophysiological recording traces of striatal MSNs in the control and in the MK801 and MK801 + CUMS groups are shown. **(E)** The average amplitude of mEPSCs in MSNs. **(F)** The total inter-event interval in MSNs is shown. **(G)** Relative visual-evoked potentials in the thalamic nuclei upon visual stimulation are shown. CUMS, chronic unpredicted mild stress; mEPSC, miniature excitatory post-synaptic current; IEI, inter-event interval; dlPFC, dorsolateral part of the prefrontal cortex; MSN, medium spiny neuron. *P < 0.05; **P < 0.01; ***P < 0.001; *N* = 5 per group.

After noticing the worsened mental and sensory functions in the schizophrenia with depression model, this model was further examined regarding whether it also exhibits unique neural mechanisms. Utilizing *in vivo* two-photon imaging, calcium concentration changes in neurons of layer 2/3 in the dorsolateral part of the prefrontal cortex (dlPFC) were recorded in awake mice which had been virally transfected with the genetically encoded calcium indicator GCaMP6s. The continuous recording of neuronal activity (Figure 3A) demonstrated that all four mouse models (MK801, CUMS, CUMS + MK801, and MK801 + CUMS) had significantly reduced activity compared to that of the untreated control mice based on the total number of calcium spikes (Figure 3B) and the frequency of calcium transients (Figure 3C). Also, the MK801 + CUMS group presented lower calcium signals compared to those of all other groups. These results largely agree with the abovementioned behavioral phenotypes, thus supporting the presence of worse pathological conditions in schizophrenia with depression.

Based on the impaired sensory gating function as shown by the lower PPI ratio in these mice (Figure 2C), the activity of the neural circuits was investigated in the thalamic nuclei, which are recognized as the integration center of sensory inputs. Using *ex vivo* patch-clamp recordings, the mEPSCs of thalamic medium spiny neurons (MSNs, Figure 3D) were evaluated. Upon comparing healthy controls to the schizophrenia (MK801) and schizophrenia with depression (MK801 + CUMS) models, MK801 administration led to elevated mEPSC amplitudes and frequencies (i.e., lower inter-event intervals), but the MK801 + CUMS group presented lower mEPSC amplitudes and frequencies (Figures 3E,F). These data indicate that the present schizophrenia with depression model may involve neural mechanisms distinct from those in schizophrenia models. As further evidence, the visual-evoked potentials in the thalamic regions in those three groups were examined. The results showed that the schizophrenia model mice had nearly normal responses to the applied visual stimuli. By contrast, the visual-evoked responses in schizophrenia with depression model mice had decreased strength. These data further support that the schizophrenia with depression model had broader and more severe neural pathway deficits than the schizophrenia model.

### Effectiveness of Antipsychotic Drugs Targeting Schizophrenia With Depressive Disorders

**FIGURE 4:**
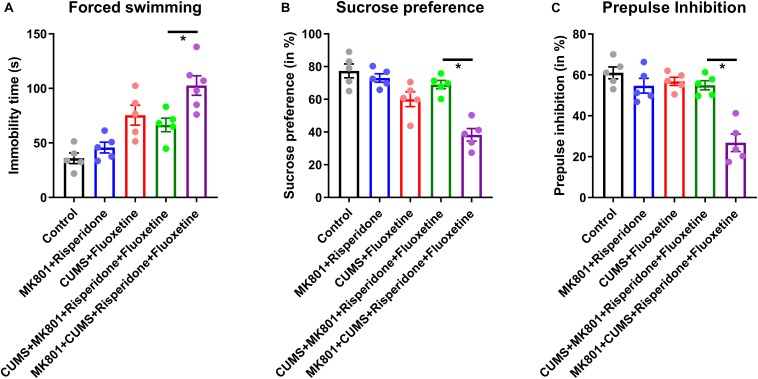
Behavioral phenotypes in mouse models of schizophrenia with depression after treatment with antidepressant and antipsychotic drugs. **(A)** Immobility time in the forced swimming test after receiving fluoxetine and/or risperidone treatment is shown. **(B)** The sucrose preference ratio in all treatment groups is shown. **(C)** PPI assay in all treatment groups is shown. CUMS, chronic unpredicted mild stress. *P < 0.05; *N* = 5–6 per group.

After acknowledging the distinct behavioral phenotype and unique neural circuit deficits in the schizophrenia with depression model, the possible pharmacological effects of commonly used antipsychotic drugs were investigated in this comorbidity model; the commonly used antidepressant fluoxetine and the antipsychotic drug risperidone were used. Pilot studies showed that both risperidone and fluoxetine had satisfactory effects in reversing schizophrenic and depressive symptoms, respectively. Theoretically, the combined use of risperidone and fluoxetine may help to relieve the comorbidity of schizophrenia and depression syndromes. However, the treatment strategy of combining these two drugs may only improve behavioral deficits in the CUMS + MK801 model. By contrast, the combination of these two drugs, following the standard protocol, did not significantly improve the immobility time in the forced swimming task (Figure 4A) and the sucrose preference ratio (Figure 4B) in the MK801 + CUMS mice. Moreover, when testing performance in the PPI test, which is one typical feature of schizophrenia, we found that, similar with those in depressive phenotypes, drug treatment rescued such sensory gating deficits in all but not in the MK801 + CUMS model (Figure 4C). These results further support the hypothesis that schizophrenia with depression has unique pathological features that are different from those of either depression or psychotic disorders. The ineffectiveness of the combined drug use suggests the necessity to develop unique drugs targeting schizophrenia with depressive disorders.

### Differential Modulation of Neural Circuit Activity by Antipsychotic Drugs

**FIGURE 5:**
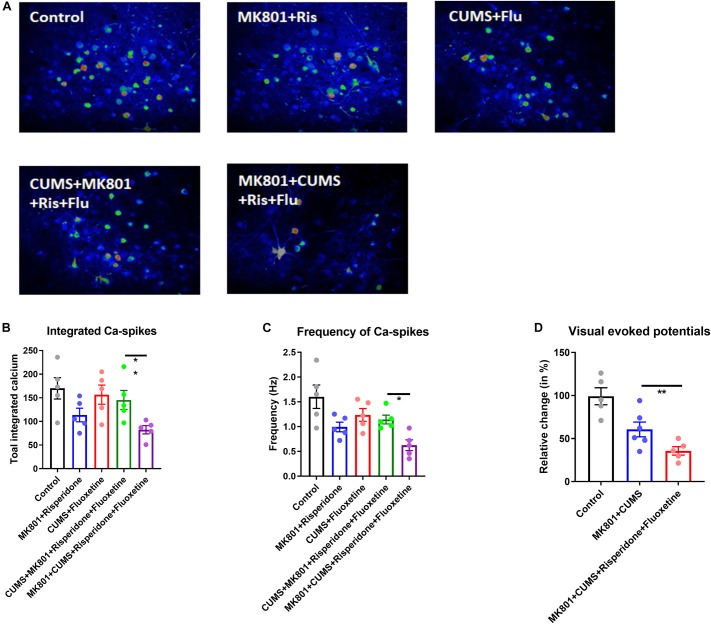
Neuronal activity of the cortical and the thalamic nuclei after drug treatment. **(A)** Representative time-lapse stacked images of L2/3 neurons in the dlPFC in all experimental groups with or without drug treatment are shown. **(B)** Integrated calcium transients over the recording time (2.5 min) are shown. **(C)** The calcium spike frequency in Hz is shown. **(D)** Visual-evoked potentials recorded from the thalamic nuclei in response to the presented stimuli are shown. Ris, risperidone; Flu, fluoxetine; CUMS, chronic unpredicted mild stress; dlPFC, dorsolateral part of the prefrontal cortex. *P < 0.05; **P < 0.01; *N* = 5–6 per group.

Based on the behavioral phenotypes and the responses to combined treatment using both antidepressant and antipsychotic drugs, the schizophrenia with depression model was theorized to exhibit distinct neural circuit activities upon drug treatment. To validate this hypothesis, *in vivo* calcium imaging was performed again in a mouse model which had received a GCaMP6s injection plus drug infusion (Figure 5A). Imaging the L2/3 neurons of the PFC revealed that an antipsychotic or antidepressant treatment can restore normal neuronal calcium signaling to a certain extent (compare Figures 3B,C with Figures 5B,C). Moreover, the combined treatment with risperidone plus fluoxetine can rescue this activity in the CUMS + MK801 mice. However, in the schizophrenia with depression model, generated by the MK801 + CUMS treatment, the combined pharmacological treatment did not achieve satisfactory effects as the calcium signaling remained at low levels after the drug treatment (Figure 5C). These data largely agree with the unchanged behavioral phenotypes as mentioned before (Figure 4). Since the data show that impaired visual-evoked potentials in thalamic nuclei occur specifically in the schizophrenia with depression model (Figure 3G), visual responses were also recorded in these mice after the drug treatment. Surprisingly, the co-treatment of risperidone plus fluoxetine further aggravated the visual response deficits instead of alleviating the dysfunction as expected (Figure 5D). These results further suggest distinct features in schizophrenia with depression, distinguishing it from other mental disorders and thus calling for novel insights into possible drug interventions.

## Discussion

In this study, a series of behavioral studies and functional recordings were performed to compare various aspects across isolated or combined models of depression and schizophrenia, followed by pharmacological treatment and *post hoc* observations. Two essential and previously unrecognized findings were established from these data, which support the holistic view on schizophrenia with depression instead of a classical comorbidity model.

First, the schizophrenia with depression model, generated by MK801 injection followed by CUMS treatment, presented more severe impairments in behavioral performance and neural activity. Specifically, the MK801 + CUMS group exhibited lower sucrose preference and PPI ratio compared to the isolated schizophrenia and depression models, respectively (Figure 2). Such model has implications in psychiatric clinicians as the comorbid of depressive symptoms in schizophrenia patients is frequently identified (Upthegrove et al., 2017), and severe depressive symptoms may further deteriorate the condition of schizophrenia patients (Hou et al., 2016). A further discussion for the potential pathological mechanisms underlying such comorbid thus has values for clinics. Although such behavioral phenotypes can also be explained by the interaction between two regulatory pathways that can further aggravate each other’s severity, a further investigation largely disagreed with this interpretation. In particular, mice of the dual-symptomatic model had impaired neuronal activity in the PFC region (Figure 3), which is critical for mental function (Hiser and Koenigs, 2018). It was further noticed that visual-evoked responses were specifically impaired in the schizophrenia with depression model (Figure 3G). These data illustrate that this dual model may involve a unique pathological mechanism distinct from that in either schizophrenia or depression alone. This mechanism may work as a top-down regulatory pathway affecting mental, sensory, and cognitive pathways across different brain regions, leading to behavioral phenotypes that share features of schizophrenia and depression.

Second, the pharmacological intervention experiments further support the view that the schizophrenia with depression model is pathologically different from the simple combination of these two mental illnesses. As suggested by Figure 4, treatment with fluoxetine or risperidone effectively relieves the typical symptoms of depression or schizophrenia, whereas the combined drug application cannot relieve the helplessness or anhedonia symptoms in the MK801 + CUMS model. Such observations in mouse models largely agree with clinical observations showing the minimal alleviation of depressive symptoms by antidepressant treatment in schizophrenia patients complicated with depressive disorders (Fond et al., 2018). This behavioral observation is further substantiated by the analysis of neuronal recordings showing that the impaired neuronal activity in the PFC cannot be relieved by the combined antipsychotic and antidepressant treatment. Taken together, this knowledge supports our conclusion that a distinct neuromodulation pathway may exist beyond the canonical mechanisms of depression or schizophrenia and lead to the dual symptoms of depression and psychotics in the present model.

Notably, the combined drug treatment did not improve the impaired sensory responses in the thalamic nuclei or the visual-evoked responses (Figure 5C). Sensory gating deficit is a prominent feature of schizophrenia and can occur in certain subpopulations of depressive patients (Micoulaud-Franchi et al., 2016). As observed in the PPI test, the MK801 + CUMS model exhibited worse sensory processing compared to the model of schizophrenia alone. Therefore, these findings provide both auditory and visual sensory functional evidence, indicating abnormalities of the neural circuits in the schizophrenia with depression model. The ineffectiveness of the combined drug treatment targeting the visual response further illustrated that antipsychotic drugs can only relieve sensory gating deficits in the classical schizophrenia model, whereas the schizophrenia with depression model has distinct neural pathways that affect the sensory processing functions.

Furthermore, different behavioral patterns exist between the CUMS + MK801 and MK801 + CUMS models; the former presents mild anhedonia, helplessness, and sensory gating deficits, whereas the latter displays aggravated behavioral deficits in both depressive and psychotic symptoms (Figure 2). Such interesting findings receive partial support from a clinical report showing the possible involvement of metabolic disorders in schizophrenia and depression, and antipsychotic treatment could further aggravate such predisposition (Kucerova et al., 2015). These differences in behavioral phenotypes were supported by calcium and electrophysiological recordings, in which the MK801 + CUMS model presented the lowest neuronal activity in the cortical or thalamic nuclei (Figure 3). These data indicate that distinct neuromodulating effects that distinguish between the CUMS + MK801 and MK801 + CUMS models exist, the former seemingly presenting additive depression and schizophrenia effects, whereas the latter may involve other mechanisms beyond these two syndromes. This hypothesis was partially supported by the pharmacological intervention assays demonstrating that the combination of typical antidepressant plus antipsychotic drugs effectively relieved symptoms in the CUMS + MK801 model but did not improve the depressive symptoms in the MK801 + CUMS model.

As a potent NMDA receptor antagonist, MK801 administration prominently affects the glutamatergic system (Thornberg and Saklad, 1996), which may further influence dopaminergic or serotoninergic systems across different brain regions (Carlsson and Carlsson, 1990). It is usually believed that patients with depression or rodent models of depression have insufficient dopaminergic/serotoninergic reward systems, leading to anhedonia and helplessness syndromes (Grace, 2016). Glutamatergic pathway remodeling may impair the resilience of dopamine or serotonin pathways, making them more vulnerable to psychological stress (Hutchison et al., 2018). Moreover, since antidepressants such as fluoxetine aim to potentiate the serotoninergic system (Perez-Caballero et al., 2014), the top-down impairment of the glutamate–serotonin system may also compromise the efficiency of such drugs, thus explaining the resilience of the MK801 + CUMS model against combined medication therapy. In summary, the seemingly paradoxical results from the CUMS + MK801 and MK801 + CUMS models support our hypothesis that schizophrenia with depression involves unique neural mechanisms, which require further dedicated studies for substantiation.

There are certain limitations in the present study. First, the sequential treatment of MK801 and CUMS in generating the psychiatric disorder model may raise the possibility that a post-schizophrenia depression model was reproduced. Such concerns can be partially relieved since the CUMS stimuli were applied when the MK801-induced psychotic symptoms still existed, rather than after their disappearance. Second, the therapeutic effect of combined medication was significantly better in the CMUS + MK801 model compared to that in the MK801 + CMUS model. These seemingly paradoxical phenotypes require further study for mechanistic substantiation. Nevertheless, major findings indicate that the glutamatergic pathway may interact with the reward system to produce the complicated symptoms in the comorbid model.

Many hypotheses have been developed in an effort to explain the neuropathology of schizophrenia and depression. Each hypothesis, however, has its bias mainly due to the research focus of the study when it was originally deployed. For example, the dopamine theory of schizophrenia mainly attempts to explain the therapeutic effect in alleviating positive symptoms (Howes and Kapur, 2009). The neurodevelopment hypothesis, on the other hand, attempts to clarify the brain damage in high-risk populations of schizophrenia (Pino et al., 2014; Murray et al., 2017). For depressive disorders, the neuroimmune theory largely fits the elevated immune factors in adolescent patients (Miller and Cole, 2012) and the increased levels of inflammatory factors that are synchronized with the acute stage of a depressive episode (Slavich and Irwin, 2014). Since previous studies have repeatedly discussed possible explanations for schizophrenia pathogenesis (Insel, 2010; Falkai and Schmitt, 2019; Sahakian and Savulich, 2019; van Rooijen et al., 2019), the present psychiatric disorder entity model of schizophrenia accompanied by depressive symptoms can explain the unfavorable prognosis of these symptoms.

To our knowledge, this is the first study to clarify the neural activity alterations in a model of schizophrenia with depression. Our animal model demonstrates that the functioning of critical neural activities in the PFC is more severely affected in schizophrenia with depression mouse models than in schizophrenia or depression model alone. More importantly, a combined treatment using antipsychotics and antidepressants cannot fully reverse either the behavioral or the neural activity impairments. These data suggest that schizophrenia complicated by depression may be a previously unrecognized mental disorder entity that is independent from other mental disorders.

## Data Availability Statement

All data generated during the current study are available from the corresponding author upon reasonable request.

## Ethics Statement

The animal study was reviewed and approved by the Animal Ethical Committee of Tianjin Medical University.

## Author Contributions

ChuaZ contributed to the conception and design of the study. ChuaZ and ChunZ conducted the experiment, collected the data and wrote the manuscript. ChunZ, DK, XZ, WW, RX, and GL performed the statistical analysis. ChunZ, YX, SL, and HT interpreted the results. All of the authors contributed to the manuscript version and read and approved the submitted version.

## Conflict of Interest

The authors declare that the research was conducted in the absence of any commercial or financial relationships that could be construed as a potential conflict of interest.
